# In memory of Professor Dr. med. Dr. h. c. Kay Brune (1941–2025), Past President of the German Society for Experimental and Clinical Pharmacology and Toxicology and honorary member of the German Society for Pharmacology

**DOI:** 10.1007/s00210-025-04837-x

**Published:** 2025-11-29

**Authors:** Burkhard Hinz, Bertold Renner, Gerd Geisslinger

**Affiliations:** 1 Institute of Pharmacology and Toxicology, Rostock University Medical Center, Schillingallee 70, 18057 Rostock, Germany; 2 Institute of Clinical Pharmacology, Technical University of Dresden, Fetscherstrasse 74, 01307 Dresden, Germany; 3 Institute of Experimental and Clinical Pharmacology and Toxicology, Friedrich-Alexander University Erlangen-Nuremberg, 91054 Erlangen, Germany; 4 Institute of Clinical Pharmacology, Goethe University Frankfurt; Fraunhofer Institute for Translational Medicine and Pharmacology; and Fraunhofer Cluster of Excellence for Immune-Mediated Diseases (CIMD), Theodor-Stern-Kai 7, 60596 Frankfurt am Main, Germany


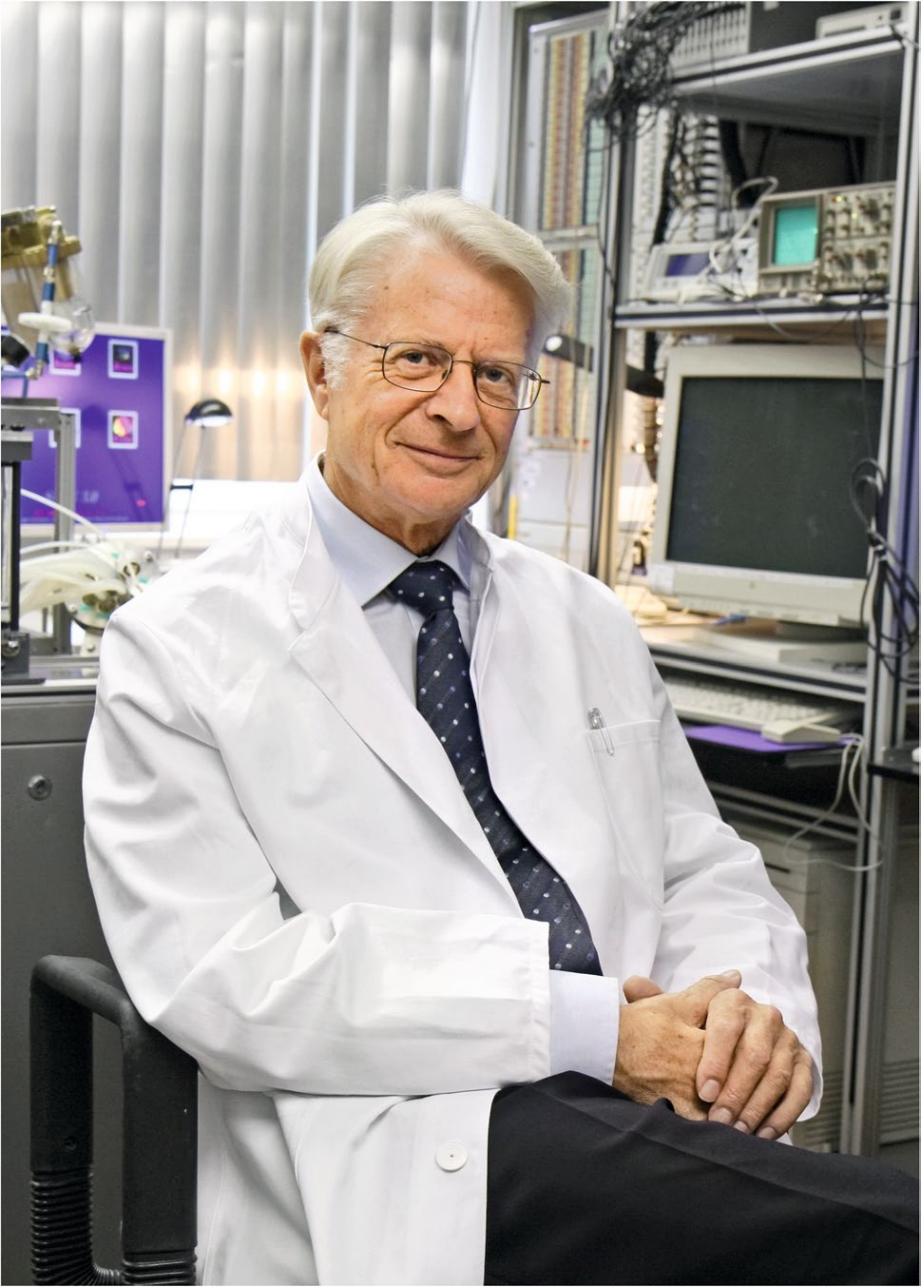
Copyright: Aufnahmen für die Stifterbroschüre der Universität Erlangen, Uni-Pressestelle, Georg Pöhlein

The German Society for Pharmacology mourns the passing of its past president and honorary member Professor Dr. med. Dr. h. c. Kay Brune, who died on 28 September at the age of 84. With his death, the pharmacological community has lost one of its most outstanding representatives and a leading pioneer and architect in pharmacological pain and inflammation research in Germany.

Kay Brune was born on 30 January 1941 in Freital (Saxony). From 1952 to 1961, he attended high school in Lüneburg (classical languages). He then studied medicine at the universities of Hamburg, Basel and Munich from 1961 to 1966, passing all his exams with distinction and being accepted into the German Academic Scholarship Foundation in 1963. He received his doctorate in 1967 in Hamburg and qualified as a specialist in pharmacology and toxicology in 1971. From 1967 to 1968, he worked as an assistant physician in Northeim (Lower Saxony), then from 1968 to 1969 and from 1970 to 1981 as an assistant, later senior physician (scientific adjunct) and private lecturer at the Biozentrum of the University of Basel (Switzerland), Department of Pharmacology. His academic teachers in Basel included Karl Bucher (pharmacology), Fritz Koller (haematology) and Georg L. Floersheim (transplantation biology). From 1969 to 1970, he was a visiting scientist at the University of North Carolina in Chapel Hill and, in 1976, a visiting professor at Wayne State University in Detroit (MI, USA).

In 1980, Kay Brune was offered a full professorship at the Department of Pharmacology, University of Kansas (Kansas City, USA) and also the Chair of Pharmacology and Toxicology at the University of Erlangen. He decided on Erlangen and, starting in 1981, established several working groups here that focused on cellular, molecular and clinical pain and inflammation research as well as computer-assisted systems for recording side effects. These efforts were soon rewarded. Together with Hermann Otto Handwerker (Erlangen) and Robert F. Schmidt (Würzburg), he successfully established the Collaborative Research Centre (SFB) “Pathobiology of pain generation and pain processing” (1992 to 2003), which was funded by the German Research Foundation. For many years, he also contributed significantly to the Collaborative Research Centers “Immunological Mechanisms in Infection, Inflammation, and Autoimmunity” and “Glaucoma, including Pseudoexfoliation Syndrome”. The achievements of the institute headed by Kay Brune were honoured with a modern new building, which was inaugurated in 1999.

This intensive research was also reflected in extensive publishing activity and in the fact that, over a period of three decades, a very large number of articles in international standard reference works (including the Handbook of Experimental Pharmacology, the Textbook of Pain and the Oxford Textbook of Pharmacology) bore the signature of Kay Brune and his academic colleagues. For almost three decades, Kay Brune himself worked as an editor of scientific journals: from 1973 to 1994 for Agents and Actions (later Inflammation Research) and from 1999 to 2001 as section editor of the Journal of Pharmacology and Experimental Therapeutics.

With his research, Professor Brune can rightly be said to have created textbook knowledge. In the 1970s, shortly after the publication of the findings by John R. Vane and colleagues (1971) that aspirin-like drugs inhibit prostaglandin synthesis, he was able to prove that it was through the persistence of non-steroidal anti-inflammatory drugs in the deep compartment of “inflammatory tissue” that sustained inhibition of inflammation and pain was achieved. These insights were followed in subsequent years by many studies by Brune’s group on the importance of pharmacokinetics in the mode of action of analgesics.

Kay Brune knew how to fascinate young people with the mechanisms of action of analgesic substances. Together with his staff of young habilitation candidates, the pharmacology of chiral analgesics was investigated, resulting, for example, in the discovery of the analgesic properties of the non-cyclooxygenase-inhibiting enantiomer R-flurbiprofen. At his chair, the mechanism of the analgesic effect of cyclooxygenase inhibitors in the dorsal horn of the spinal cord was identified. Furthermore, it was proven that paracetamol is a preferential cyclooxygenase-2 inhibitor, which led to a delayed explanation of various pharmacological properties of this old analgesic. In the final years of his active career, he was involved in work on imaging hyperalgesia using functional magnetic resonance imaging. Many of his former habilitation graduates now hold professorships and chairs in pharmacology (e.g. in Frankfurt am Main, Zurich and Rostock) or leading positions in the pharmaceutical industry.

In connection with his research, Kay Brune’s strong commitment to constructive animal welfare must also be mentioned. In this spirit, he served as chairman of the board of trustees of the Doerenkamp-Zbinden Foundation from 1995 to 2004. The aim of this foundation is to plan animal experiments that are indispensable in biomedical research in accordance with the ethical 3R concept (replacement, refinement, reduction). In recognition of this work, Professor Brune was given the opportunity to spend the last years of his professional life conducting research as the holder of a Doerenkamp-Zbinden professorship for innovation in animal and consumer protection until mid-2012.

Throughout his active professional life, Kay Brune received numerous prestigious honours. These include, just to mention a few, the Pappenheim Prize from the German Society for Haematology (1972), the German Pain Prize (1996), the Felix Wankel Special Prize for Animal Welfare Research (2002), the Sertürner Prize (2007) and an honorary doctorate from the University of Timișoara (1994).

The list of honorary positions held by Kay Brune is also extensive: President of the International Association of Inflammation Societies (1995 to 1998), Vice-President of the German Society for the Study of Pain (1996 to 1999), appointment to the Scientific Committee of the Health Research Council of the Federal Ministry of Education and Research (1997), appointment to the Senate Committee for Collaborative Research Centres of the German Research Foundation and, finally, election as President of the German Society for Experimental and Clinical Pharmacology and Toxicology (1999). During his last mentioned presidency (2000 to 2002), the executive committee and sections worked together to develop new statutes that granted autonomy to all sub-societies (experimental pharmacology, clinical pharmacology, toxicology) in their specific areas of expertise. The cohesion of the association as a whole was strengthened through pre-symposia and joint events with other associations, increased public relations work and publications. In recognition of his outstanding merits to this society, Professor Brune was awarded honorary membership of the German Society for Pharmacology in 2016.

Professor Brune’s work in pharmacology has set exceptionally high standards in research, teaching and voluntary work, which have had a lasting impact on our discipline and will continue in the future.

We bow in gratitude to a great pharmacologist, teacher and person.

On behalf of his many former academic associates:

Burkhard Hinz, Bertold Renner and Gerd Geisslinger.

